# Dark Empaths and Intimate Partner Violence: A Latent Profile Analysis of Dark Tetrad Traits and Empathy Dimensions in Face-to-Face and Cyber Violence Perpetration

**DOI:** 10.3390/bs16101780

**Published:** 2026-09-29

**Authors:** Manuel Galán, Niklas Gerber, Lydia Valls, Sergio Molina, David Pineda

**Affiliations:** 1 Department of Psychology, Catholic University of Murcia, 30107 Guadalupe, Spain; mgalan@ucam.edu (M.G.); nmgerber@alu.ucam.edu (N.G.); 2 Department of Health Psychology, Miguel Hernandez University of Elche, 03202 Elche, Spain; smolina@umh.es (S.M.); dpineda@umh.es (D.P.)

**Keywords:** dark tetrad, dark empath, empathy, IPV, latent profile analysis

## Abstract

Intimate partner violence (IPV) has been consistently linked to Dark Tetrad traits (narcissism, Machiavellianism, psychopathy, and sadism), typically associated with reduced empathy. However, cognitive empathy may be used instrumentally rather than protectively, giving rise to a “dark empath” profile—elevated dark traits alongside preserved empathetic mechanisms—which remains understudied in IPV perpetration. The present study used latent profile analysis to identify personality profiles based on Dark Tetrad traits and empathy and examined differences in face-to-face and cyber IPV perpetration. Participants (*N* = 324; 70.4% women; 82.1% based in Spain) completed measures of the Dark Tetrad, empathy, and face-to-face and cyber IPV perpetration. Four profiles emerged: Empathic Non-Dark, Typical, Dark Cognitive Empath, and Dark Non-Empathic. The Dark Non-Empathic profile showed the clearest elevations in face-to-face physical and psychological violence. For cyber monitoring/control, the Dark Cognitive Empath profile showed the highest estimated score and was significantly higher than the Empathic Non-Dark and Typical profiles, but did not differ significantly from the Dark Non-Empathic profile. These findings provide preliminary evidence that preserved cognitive empathy alongside elevated dark traits may be associated with elevated cyber monitoring/control, although this pattern was not unique to the Dark Cognitive Empath profile. The findings further suggest that cognitive and affective empathy should be distinguished when examining IPV perpetration.

## 1. Introduction

Intimate partner violence (IPV) constitutes one of the most prevalent and damaging forms of interpersonal aggression worldwide. The World Health Organization (2021) estimates that approximately one in three women globally has experienced physical and/or sexual violence perpetrated by an intimate partner, with rates rising when psychological forms of abuse are included. Beyond its high prevalence, IPV carries severe and often long-lasting consequences for victims, including depression, post-traumatic stress disorder, anxiety, reduced self-esteem, and impaired social functioning (World Health Organization, 2021). These consequences underscore the necessity of identifying the psychological risk factors that underlie its perpetration. IPV, however, takes many forms: it encompasses physical, sexual, and psychological aggression, as well as economic abuse and, increasingly, digital forms of violence enacted through electronic devices and social media platforms commonly referred to as cyber intimate partner violence (Borrajo et al., 2015). Although face-to-face and cyber forms of IPV may share underlying relational dynamics, the psychological factors driving each and the mechanisms through which they unfold are not necessarily identical, a distinction the present study sets out to explore.

### 1.1. Dark Tetrad Traits and Intimate Partner Violence

Among the individual-level factors associated with IPV perpetration, dark personality traits have attracted substantial research attention (e.g., Pineda et al., 2022). The Dark Triad, a constellation of subclinical traits composed of narcissism, Machiavellianism, and psychopathy, was first formally conceptualized by Paulhus and Williams (2002) as a cluster of socially aversive yet non-clinical personality patterns sharing a common core of callousness and interpersonal antagonism. This model was subsequently extended by Chabrol et al. (2009) with the addition of everyday sadism, yielding the Dark Tetrad. Subclinical narcissism is characterised by inflated self-appraisal, a pronounced need for admiration, and a sense of entitlement (Campbell & Miller, 2011; Raskin & Hall, 1981). This grandiose presentation is typically distinguished from vulnerable narcissism, which reflects underlying insecurity, hypersensitivity to criticism, and defensiveness masked by a fragile self-image (Wink, 1991). Machiavellianism reflects a cynical, strategically exploitative orientation in which the manipulation of others is regarded as an acceptable means to personal ends (Christie & Geis, 1970; Jonason & Krause, 2013). Subclinical psychopathy is defined by callousness, reduced emotional responsiveness, heightened impulsivity, and a propensity for rule-breaking and interpersonal manipulation (Hare, 1999; Paulhus & Williams, 2002). This is often further divided into primary psychopathy, marked by low anxiety and instrumental, unemotional manipulation, and secondary psychopathy, characterised by impulsivity, emotional reactivity, and anxiety-driven aggression (Levenson et al., 1995), although it is also commonly studied unidimensionally (Jones & Paulhus, 2014). Everyday sadism captures the tendency to derive pleasure from inflicting harm on or observing the suffering of others in non-criminal, quotidian contexts (Chabrol et al., 2009). Several of these traits have individually been linked to antisocial outcomes; psychopathic and sadistic traits to juvenile delinquency (Chabrol et al., 2009), and psychopathy specifically to violent crime and recidivism (Hare, 1999). Additionally, their role as risk factors for IPV perpetration has been empirically supported across multiple study designs and populations (Carton & Egan, 2017; Carter & Egan, 2022; Cascardi et al., 2025).

More specifically, the links between each dark trait and specific forms of IPV are both robust and trait-specific. Psychopathy and sadism emerge as the strongest predictors of physical aggression within intimate relationships, given their shared features of callousness, impulsivity, and the absence of remorse (Carton & Egan, 2017). Machiavellianism is more consistently associated with non-physical forms of abuse, particularly psychological and cyber forms, reflecting its strategic, premeditated character. Narcissism, while less consistently linked to physical violence, is associated with reactive aggression in response to perceived ego-threats and with controlling behaviour in romantic relationships (Campbell & Miller, 2011; Carton & Egan, 2017). In the digital sphere, Pineda et al. (2022) showed that psychopathy and narcissism were the strongest predictors of cyber controlling behaviour, while sadism was primarily associated with cyber IPV victimisation. Machiavellianism showed a negative association with direct cyber aggression attributed to the trait’s characteristically cautious, risk-averse strategic orientation. More broadly, the Dark Tetrad has been shown to predict IPV perpetration beyond general personality dimensions, with psychopathy emerging as the most robust individual predictor across both physical and psychological subtypes (Carter & Egan, 2022).

### 1.2. Empathy: Dimensions and Their Relationship with Dark Traits

Central to the understanding of why dark personality traits predispose individuals toward harmful interpersonal behaviour is the construct of empathy, which is the capacity to understand and share the emotional states of others, widely regarded as a critical inhibitor of aggression and a facilitator of prosocial behaviour (Davis, 1983; Hoffman, 2000). The two-dimensional model distinguishing between cognitive empathy (the capacity to understand others’ emotional states without being personally affected by them) and affective empathy (the capacity to connect emotionally with another person’s feelings) has become the most widely adopted framework in personality and violence research (e.g., Cuff et al., 2016; Decety & Jackson, 2004; Jolliffe & Farrington, 2006). Whereas affective empathy is broadly understood as a direct inhibitor of harmful behaviour, cognitive empathy is a value-neutral capacity that can serve either prosocial or exploitative ends depending on the motivational context in which it is deployed (Heym et al., 2019; Wai & Tiliopoulos, 2012).

This context-dependence is especially apparent when examining the empathic profiles of individuals high in dark personality traits, which reveal a nuanced picture: The conventional assumption that dark personalities are overall empathy-deficient has been significantly qualified by studies employing the cognitive-affective distinction (Jonason & Krause, 2013; Wai & Tiliopoulos, 2012). The most consistent finding is that dark traits, particularly psychopathy and Machiavellianism, are primarily associated with deficits in affective empathy, while cognitive empathy is comparatively spared or even enhanced in certain configurations. Sadism has been shown to relate negatively to both cognitive and affective empathy, positioning it as the trait most broadly implicated in emotional unresponsiveness toward others’ suffering (Heym et al., 2019; Jonason & Krause, 2013; Wai & Tiliopoulos, 2012). These patterns suggest that the more relevant question is not whether a dark personality individual has empathy, but what kind of empathy they have and what they do with it (Diller et al., 2026). This distinction remains a point of discussion in the literature as research treating empathy deficits as a unitary marker of dark personality has tended to ignore the divergence between cognitive and affective empathy shown in profile-based work; this divergence is central to the rationale of the present study.

### 1.3. The Dark Empath Profile and Its Relevance to IPV

The observation that some individuals combine elevated dark traits with intact or above-average empathic capacities gave rise to the empirical identification of the dark empath profile. Heym et al. (2021) were the first to formally characterise this profile using latent profile analysis (LPA) on a sample of 991 participants, in which cognitive and affective empathy were parsed into separate indicators alongside the Dark Triad Traits. Their analysis identified four distinct groups: the traditional Dark Triad cluster (high dark traits, low empathy), the Dark Empath (high dark traits, high empathy), Empaths (low dark traits, high empathy), and Typicals (low dark traits, average empathy). Critically, dark empaths displayed higher extraversion and agreeableness and better wellbeing than traditional Dark Triad individuals yet maintained an antagonistic core comparable to that of the traditional Dark Triad cluster in terms of vulnerable narcissism, secondary psychopathy, and Machiavellian facets. A subsequent study by Bloxsom et al. (2021) found that dark traits combined with empathy showed a specific influence on attachment patterns in women, suggesting that the dark empath profile has particular relevance in intimate relational contexts. Together, these findings challenge the longstanding assumption that empathy functions as a universally protective personality feature.

The potential implications of the dark empath profile for intimate partner violence have not yet been directly examined. In close relational contexts, the combination of dark traits with preserved or elevated cognitive empathy may give rise to particularly sophisticated, covert, and difficult-to-detect forms of abuse. Cognitive empathy may function as an instrument of strategic control when it operates in the absence of affective resonance. This dynamic may manifest with particular intensity in the digital sphere, where physical absence and reduced immediate social feedback lower the inhibitory cues that might otherwise constrain harmful behaviour (Pineda et al., 2022). Unlike face-to-face violence, where psychopathy and sadism tend to be the strongest predictors, online environments may be especially favourable to the more calculated, instrumentally empathic aggression that the dark empath profile implies. Furthermore, the apparent social competence and emotional awareness of dark empaths may make their abusive behaviour harder for victims to recognise and harder for professionals to identify in assessment contexts (Bloxsom et al., 2021; Heym et al., 2021). This has significant implications for forensic assessment and the design of reintegration programmes: interventions aimed at increasing empathic capacity in IPV perpetrators may paradoxically enhance the manipulative potential of individuals with this profile (Cascardi et al., 2025).

### 1.4. Study Rationale; Aims and Hypotheses

Despite the growing body of evidence linking the Dark Tetrad to IPV and the more recent emergence of the dark empath as a theoretically compelling personality profile, the intersection of these two studies remains unexplored. Existing research has predominantly employed variable-centred approaches, examining the independent or additive contributions of dark traits and empathy dimensions to IPV outcomes, without considering how specific combinations of these characteristics co-occur within individuals (Howard & Hoffman, 2018). A person-centred approach, such as latent profile analysis, addresses this limitation by allowing for the identification of naturally occurring subgroups that differ in the overall pattern of trait combinations (Heym et al., 2021). However, Heym et al. (2021) derived their profiles using only the Dark Triad, without the sadism component of the Dark Tetrad, and did not examine any interpersonal violence outcome. Given that sadism has shown distinct associations with both empathy and IPV perpetration, one cannot assume Heym’s Dark Triad-based profiles would generalise unchanged to a Tetrad model with an IPV focus. Additionally, no study to date has directly investigated whether the dark empath profile is associated with differential patterns of IPV perpetration, and specifically whether this profile shows heightened engagement in online forms of violence compared to face-to-face aggression. The present study addresses this gap by using LPA to identify personality profiles based on Dark Tetrad traits and both cognitive and affective empathy dimensions, and by examining whether these profiles differ in their perpetration of face-to-face and cyber IPV.

Building on this rationale, the objective of the present manuscript is to examine how Dark Tetrad traits and affective and cognitive empathy relate to the perpetration of face-to-face and cyber intimate partner violence, as well as to identify latent personality profiles that combine these dimensions. Based on prior variable-centred research, we expect affective empathy to correlate negatively with dark traits, particularly psychopathy, Machiavellianism, and sadism, while cognitive empathy is expected to show weaker or negligible associations with these traits, which is consistent with the evidence that cognitive empathy is comparatively preserved in dark personalities (H1). This variable-level pattern provides the empirical basis for the person-centred approach adopted in the present study because if dark traits and cognitive empathy are largely independent at the variable level, this raises the possibility that some individuals combine elevated dark traits with preserved cognitive empathy, meaning the emergence of distinct personality profiles (H2). A correlational analysis alone cannot identify this configuration, whereas latent profile analysis is specifically suited to detecting such configurations. Lastly, we expect these profiles to differ significantly in their perpetration of IPV, with profiles characterised by higher dark traits and lower empathy showing a greater propensity toward violent behaviour (H3).

## 2. Materials and Methods

### 2.1. Participants and Procedure

The sample comprised 324 participants, including 228 women (70.37%) and 96 men (29.63%), aged between 18 and 87 years (*M* = 26.68, *SD* = 12.70). Most participants were from Spain (82.10%), followed by Mexico (6.5%) and Panama (1.5%), with smaller proportions from a variety of other countries. Regarding relationship status, the majority were in an unmarried partnership (53.43%), while 26.23% were single and 12.04% were married. Among participants who were currently in a relationship (*n* = 231), the most common relationship duration was between 1 and 3 years (38.50%), followed by less than 1 year (22.10%), more than 15 years (15.60%) between 3 and 6 years (12.60%), and between 6 and 15 years (11.30%). With respect to relationship length, most participants had been with their partner between 0 and 3 years (43.27%), while only 11.11% had been in their relationship for more than 15 years. The inclusion criteria were: (1) being 18 years of age or older, (2), providing informed consent to participate in the study, and (3) completing the entire online survey.

After the Ethics Committee of the Universidad Católica de Murcia (CE012517) approved the research, a cross-sectional online survey was administered using LimeSurvey (Version 5.6.11), hosted on a secure university server that complies with European Union requirements regarding data security and personal data protection. The survey link was disseminated via WhatsApp among personal contacts and acquaintances of the research team, following a snowball sampling approach. WhatsApp was used solely to distribute the survey link; no study data or participant responses were collected or stored through WhatsApp. In turn, those who responded were also asked to share the survey with their own contacts. Before beginning, participants were presented with a participant information sheet within LimeSurvey describing the study’s aims, the requirement of being of legal age, the study’s ethical approval, confidentiality, and their right to withdraw at any time without consequences. After reading this information, participants were required to click a button to confirm that they had read and understood the information provided and to explicitly provide their informed consent to participate in the study. Only participants who provided this electronic informed consent were able to proceed with the survey. Participants did not receive compensation for completing the survey.

### 2.2. Instruments

**Short Dark Triad** (SD3; Jones & Paulhus, 2014)

The Spanish version of the SD3 (Pineda et al., 2020) was used to evaluate the Dark Triad traits. It is a 27-item instrument measuring subclinical narcissism, Machiavellianism, and psychopathy using a 5-point Likert scale (0 = Strongly Disagree to 4 = Strongly Agree), with 9 items per dimension. Example items include: “many group activities tend to be dull without me” (narcissism); “it’s not wise to tell your secrets” (Machiavellianism); and “I avoid dangerous situations” (psychopathy). Reliability for the current sample: narcissism, α = 0.65, ω = 0.65; Machiavellianism, α = 0.77, ω = 0.77; psychopathy, α = 0.71, ω = 0.72.

**Assessment of Sadistic Personality** (ASP; Plouffe et al., 2017)

The Spanish version of the ASP (Pineda et al., 2021) is a 9-item instrument assessing everyday sadism using a 5-point Likert scale (0 = Strongly Disagree to 4 = Strongly Agree). An example item is: “Being mean to others can be exciting.” The Spanish version has demonstrated adequate psychometric properties in combination with the SD3 (Pineda et al., 2021). Reliability for the current sample: α = 0.80, ω = 0.81.

**Basic Empathy Scale** (BES; Jolliffe & Farrington, 2006)

The Spanish version of the BES (Villadangos et al., 2016) was used to assess empathy. It is a 20-item instrument measuring two dimensions—cognitive empathy and affective empathy—using a Likert-type scale. Reliability for the current sample: cognitive empathy, α = 0.73, ω = 0.73; and affective empathy, α = 0.82, ω = 0.82.

**Revised Conflict Tactics Scales** (CTS2; Straus et al., 1996)

The Spanish version of the CTS2 (Loinaz et al., 2012) was used to assess IPV perpetration. It is a 78-item instrument organised into five subscales—physical violence, sexual violence, psychological violence, and negotiation—using a frequency-based response format. For the purposes of the present study, only the perpetration items were used, and negotiation was retained as a non-violent comparison subscale; victimisation items were not analysed. Reliability for the current sample: physical violence, α = 0.68, ω = 0.70; sexual violence, α = 0.12; psychological violence, α = 0.72, ω = 0.74; and negotiation, α = 0.88, ω = 0.87.

**Cyber Dating Abuse Questionnaire** (CDAQ; Borrajo et al., 2015)

The CDAQ (Borrajo et al., 2015) was used to assess cyber IPV. It is a 40-item instrument that measures cyber IPV, evaluating direct aggression and monitoring/control behaviours—rated on a 6-point frequency scale ranging from never to always. Consistent with the CTS2, only perpetration items were analysed, while victimisation items were excluded. The perpetration items were analysed separately as Direct Aggression (11 items) and Monitoring/Control (9 items), rather than combined into a total score. Example items include: “I wrote a comment on the wall of a social network to insult or humiliate my partner or former partner” (Direct Aggression) and “My partner or ex-partner has controlled the time of my last connection to mobile applications” (Borrajo et al., 2015). In the present sample, Monitoring/Control showed good internal consistency (α = 0.83, ω = 0.84), whereas Direct Aggression showed low internal consistency (α = 0.36). Consequently, Direct Aggression was retained in the analyses for completeness but was not substantively interpreted.

### 2.3. Data Analysis

Descriptive statistics, reliability estimates (Cronbach’s α), and bivariate correlations were calculated using Mplus (Version 8.6; Muthén & Muthén, 2017). Latent profile analysis (LPA) was conducted to identify distinct subgroups of participants based on their scores on the four Dark Tetrad traits and the two empathy dimensions. Prior to the analysis, the six profile indicators were standardised. Models from one to six profiles were estimated using robust maximum likelihood estimation (MLR). Indicator means were freely estimated across profiles, while within-profile variances were constrained to be equal across profiles and indicator covariances were fixed to zero. To reduce the risk of local maxima, multiple random starts were used, and the retained four-profile solution was re-estimated using 500 random sets of starting values, with 100 retained for final-stage optimisation. Replication of the best loglikelihood was examined. The Bayesian Information Criterion (BIC), Akaike Information Criterion (AIC), sample-size adjusted BIC (SSA-BIC), entropy, Vuong-Lo-Mendell-Rubin test (VLMR), and adjusted Lo-Mendell-Rubin likelihood ratio test (adjusted LRT) were considered when comparing alternative profile solutions. Lower AIC, BIC, and SSA-BIC values, higher entropy, significant likelihood ratio tests, profile size, model stability, parsimony, and theoretical interpretability were considered jointly when selecting the retained solution (Spurk et al., 2020). The resulting profile solution was interpreted according to the relative levels of dark traits and empathy characterising each profile.

Differences in IPV perpetration across the retained profiles were subsequently examined in Mplus using the Bolck-Croon-Hagenaars (BCH) auxiliary-variable procedure. This approach accounts for uncertainty in latent profile classification while preventing the distal outcomes from influencing the formation of the profiles. For the CDAQ, direct aggression and monitoring/control were analysed separately. For the CTS2, physical violence, sexual violence, psychological violence, and negotiation were analysed as separate outcomes. BCH-adjusted means and standard errors were estimated for each profile, and overall and pairwise Wald chi-square tests were used to examine differences between profiles. CDAQ direct aggression and CTS2 sexual violence were retained in the analyses and reported for completeness, but were not substantively interpreted because of their low internal consistency in the present sample. Given the marked positive skewness of several IPV outcomes, sensitivity analyses were conducted by repeating the BCH comparisons using log-transformed scores. These analyses were used to examine whether the pattern of profile differences was robust to non-normality and extreme observations.

## 3. Results

Table 1 presents the means, standard deviations, and bivariate correlations for all study variables. For descriptive context, the mean SD3 scores observed in the present sample (narcissism: *M* = 16.21, *SD* = 5.26; Machiavellianism: *M* = 17.07, *SD* = 6.10; psychopathy: *M* = 8.87, *SD* = 4.93) were somewhat higher than those reported in the Spanish validation sample using the same instrument (*M* = 14.83, *SD* = 4.63; *M* = 15.01, *SD* = 5.58; and *M* = 7.61, *SD* = 4.84, respectively; Pineda et al., 2020), suggesting that the present sample was not characterised by unusually low levels of Dark Triad traits.

The four Dark Tetrad traits were positively and moderately to strongly intercorrelated (*r*s = 0.28 to 0.71), with the strongest overlap between psychopathy and Machiavellianism (*r* = 0.65) and between psychopathy and sadism (r = 0.71). Affective empathy correlated negatively with all four dark traits (*r*s = −0.14 to −0.27), consistent with its role as a general inhibitor of dark personality expression, whereas cognitive empathy showed negligible associations with the Dark Tetrad traits (*r*s = −0.03 to 0.03) and only a moderate positive correlation with affective empathy (*r* = 0.34). Regarding IPV perpetration, Machiavellianism, psychopathy, and sadism were positively associated with both cyber violence (CDAQ Cyber Violence) and face-to-face perpetration (CTS2 Physical Violence and CTS2 Psychological Violence; *r*s = 0.14 to 0.33), while narcissism showed weaker, more inconsistent associations. Affective empathy was negatively associated with CTS2 Physical and Sexual Violence (*r*s = −0.18 to −0.14) but essentially unrelated to cyber violence. Cognitive empathy showed small positive correlations with CDAQ Cyber Violence, CTS2 Psychological Violence, and CTS2 Negotiation (*r*s = 0.11 to 0.19). Overall, these findings provided partial support for H1. The predicted negative associations were consistently observed for affective empathy, whereas cognitive empathy was essentially unrelated to the Dark Tetrad traits. The predicted positive associations with IPV perpetration were also more consistently observed for Machiavellianism, psychopathy, and sadism than for narcissism, which showed weaker and less consistent associations with IPV outcomes.

**Figure 1:**
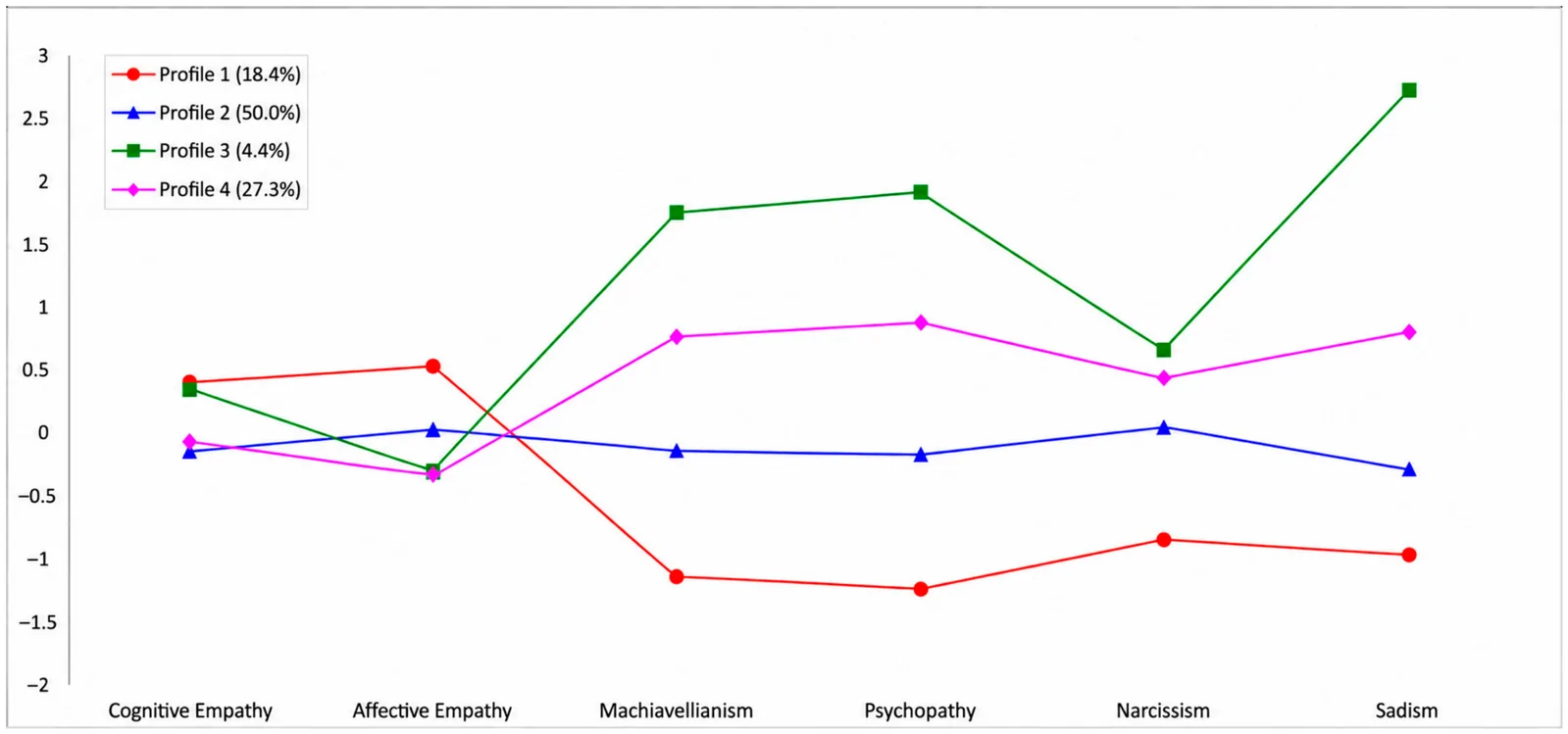
Mean values of cognitive and affective empathy and the dark tetrad traits as a function of the latent profile analysis.

Regarding the latent profile analysis (LPA), Table 2 shows the fitting values for the solutions of 1 to 6 different profiles. Considering the fit indices, classification accuracy, profile size, parsimony, and theoretical interpretability, the four-profile solution was retained, partially confirming H2. This solution showed good classification accuracy (entropy = 0.832), and both the VLMR (*p* = 0.0459) and adjusted LRT (*p* = 0.0480) supported improvement over the three-profile solution. The best loglikelihood value was replicated across random starts. Average posterior probabilities for the most likely profile membership were 0.910 for the Empathic Non-Dark profile, 0.899 for the Typical profile, 0.951 for the Dark Cognitive Empath profile, and 0.904 for the Dark Non-Empathic profile. Although the AIC and SSA-BIC continued to decrease in the five-profile solution, the BIC increased, and neither the VLMR nor the adjusted LRT supported the addition of a fifth profile. Moreover, the five-profile solution included an additional profile containing only two participants and showed estimation problems. The six-profile solution similarly produced a two-participant profile, did not receive support from the likelihood ratio tests, and showed problems with model stability and estimation. Thus, the statistical evidence did not consistently favour the more complex solutions and, considering model fit together with profile size, stability, parsimony, and interpretability, the four-profile solution was retained as the most parsimonious and stable representation of the data. This solution (Figure 1) was characterised as follows: Profile 1 (P1; *n* = 56; 10.70% men): High empathy and low dark traits—Empathic Non-Dark; Profile 2 (P2; *n* = 166; 33.10% men): Average empathy and average dark traits—Typical; Profile 3 (P3; *n* = 14; 14.30% men): High cognitive empathy and high dark traits—Dark Cognitive Empath; Profile 4 (P4; *n* = 88; 37.50% men): Low empathy and high dark traits—Dark Non-Empathic. Profile membership differed significantly as a function of sex, χ^2^(3) = 14.78, *p* = 0.002, Cramér’s *V* = 0.21. Despite its small size, the Dark Cognitive Empath profile showed high classification accuracy, with an average posterior probability of 0.951. In addition, comparison with the three-profile solution showed that all 14 participants assigned to this profile originated from the broader dark-trait profile, suggesting that the fourth profile represented an interpretable subdivision of this group.

Distributional diagnostics indicated substantial positive skewness for several IPV outcomes, particularly CDAQ direct aggression (skewness = 4.18, kurtosis = 24.49), CTS2 physical violence (skewness = 4.14, kurtosis = 19.69), and CTS2 sexual violence (skewness = 4.92, kurtosis = 23.93). CDAQ monitoring/control (skewness = 1.84, kurtosis = 3.72) and CTS2 psychological violence (skewness = 1.31, kurtosis = 1.25) showed more moderate positive skewness, whereas negotiation was comparatively symmetric (skewness = −0.51, kurtosis = −0.54). Profile differences in IPV outcomes were therefore examined using the BCH procedure, with additional log-transformed BCH analyses conducted as a sensitivity check.

Table 3 presents the BCH-adjusted means and comparisons across the four profiles. Significant overall profile differences were observed for both CDAQ dimensions and for CTS2 physical, sexual, and psychological violence, whereas no differences were observed for negotiation. CDAQ direct aggression and CTS2 sexual violence were retained in the table for consistency but were not interpreted because of their low internal consistency. For CDAQ monitoring/control, the Dark Cognitive Empath profile showed the highest estimated score and was significantly higher than the Empathic Non-Dark and Typical—Average Empathy and Dark Traits—profiles, but did not differ significantly from the Dark Non-Empathic profile. The Dark Non-Empathic profile also showed significantly higher monitoring/control than the Empathic Non-Dark and Typical profiles.

For face-to-face IPV, the Dark Non-Empathic profile showed the highest estimated level of physical violence and differed significantly from the Empathic Non-Dark and Typical profiles. The Dark Cognitive Empath profile did not differ significantly from the Typical or Dark Non-Empathic profiles on physical violence. For psychological violence, the Dark Cognitive Empath and Dark Non-Empathic profiles showed similarly elevated scores and did not differ significantly from one another. The Dark Cognitive Empath profile was significantly higher than the Empathic Non-Dark profile, whereas the Dark Non-Empathic profile was significantly higher than both the Empathic Non-Dark and Typical profiles. No profile differences were found for negotiation. Overall, this pattern provided partial support for H3. Although profiles characterised by elevated dark traits generally showed greater IPV perpetration, the Dark Cognitive Empath profile showed the highest estimated level of cyber monitoring/control despite its preserved cognitive empathy, revealing a differentiation between cyber and face-to-face IPV that was not fully anticipated by H3.

Sensitivity analyses using log-transformed IPV scores showed a comparable overall pattern of profile differences, supporting the robustness of the main BCH findings. Demographic composition differed across profiles for age, sex, relationship status, and relationship duration, but not for country. Sensitivity analyses adjusting the BCH distal-outcome comparisons separately for age, sex, and relationship status did not materially alter the pattern of IPV differences across profiles.

## 4. Discussion

The main aim of the present study was to examine whether profiles based on Dark Tetrad traits and empathy were associated with different patterns of IPV perpetration across face-to-face and cyber contexts. The latent profile analysis identified four distinct groups: Empathic Non-Dark, Typical, provisionally labelled Dark Cognitive Empath, and Dark Non-Empathic, consistent with H2. Overall, the Empathic Non-Dark reported the lowest levels of violent behaviour, whereas the Dark Cognitive Empath and Dark Non-Empathic profiles showed different forms of elevated perpetration. Across the face-to-face measures, the clearest distinction between profiles emerged for psychological violence: both the Dark Non-Empathic and Dark Cognitive Empath profiles reported significantly more psychological violence than the Empathic Non-Dark and Typical profiles, without differing significantly from one another, suggesting that elevated dark traits may be particularly relevant to psychologically aggressive behaviours regardless of whether cognitive empathy is preserved. Psychological aggression may offer a means through which manipulative, hostile, or coercive tendencies associated with dark personality traits are expressed within intimate relationships without necessarily escalating to overt physical aggression (Carter & Egan, 2022; Carton & Egan, 2017). The Dark Non-Empathic profile also reported significantly more physical violence than the Empathic Non-Dark and Typical profiles, showing the clearest overall pattern of face-to-face physical aggression; the Dark Cognitive Empath profile did not differ significantly from the other groups on this measure. The CTS2 Sexual Violence subscale was retained in the analyses and reported for transparency, but given its extremely low internal consistency in the present sample (α = 0.12), results involving this subscale are not interpreted substantively. No differences emerged for negotiation, indicating that the profiles were distinguished primarily by aggressive behaviours rather than by constructive conflict-resolution strategies.

Turning to the pattern of associations among the study variables, cognitive empathy showed no significant association with any of the four Dark Tetrad traits (*rs* = −0.03 to 0.03), indicating that possessing dark personality traits does not, on its own, suppress the capacity to understand others’ mental states. This independence is what makes the coexistence of elevated dark traits and preserved cognitive empathy—the dark empath configuration—psychologically plausible rather than contradictory (Heym et al., 2019, 2021). Affective empathy, in contrast, related negatively to all four traits, in line with its established role as a broad inhibitor of antisocial tendencies. Taken together, these findings provide partial support for H1. The predicted negative associations were supported for affective empathy but not for cognitive empathy, which was essentially unrelated to the Dark Tetrad traits. In addition, the predicted positive associations with IPV perpetration were more consistent for Machiavellianism, psychopathy, and sadism than for narcissism. Among the dark traits, sadism showed a comparatively stronger connection to the cyber form of aggression than to face-to-face violence, hinting at a preference for indirect, distance-mediated expressions of harm consistent with the profile differences reported below.

These findings are consistent with, and extend, the broader empathy–dark personality literature. Prior work has repeatedly shown that dark traits are consistently and negatively related to affective empathy, while their relationship with cognitive empathy or other related constructs has been far less consistent—sometimes null, sometimes even positive (Heym et al., 2019; Rico-Bordera et al., 2025; Wai & Tiliopoulos, 2012). The emergence of the Dark Cognitive Empath profile in the present sample, as a subgroup combining elevated dark traits with intact cognitive empathy but no comparable elevation in affective empathy, may offer a plausible explanation for this inconsistency. Rather than reflecting measurement noise, the mixed findings on cognitive empathy across studies may partly reflect a genuine dark empath subgroup nested within the broader dark-trait population, one whose empathic capacity is selectively preserved rather than uniformly impaired (Heym et al., 2021). Given the small size of this profile in the present sample, however, the Dark Cognitive Empath label should be regarded as provisional and descriptive rather than as evidence of a stable personality type.

Furthermore, as stated, the present findings provide preliminary evidence connecting the dark empath configuration with IPV perpetration, offering partial support for H3. For cyber monitoring/control behaviour—the only CDAQ subscale substantively interpreted here, given the low reliability of the Direct Aggression items (α = 0.36)—the Dark Cognitive Empath profile showed the highest estimated score among the four profiles. After accounting for classification uncertainty, this profile differed significantly from the Empathic Non-Dark and Typical profiles, but not from the Dark Non-Empathic profile; sensitivity analyses using log-transformed scores showed the same overall pattern. This suggests that elevated monitoring/control behaviour is not exclusive to individuals with preserved cognitive empathy, but is shared with the broader dark-trait-with-low-empathy profile, although the small size of the Dark Cognitive Empath profile (*n* = 14) warrants caution when interpreting this non-significant difference. In contrast, the Dark Non-Empathic profile was more clearly distinguished by face-to-face psychological and physical aggression. This pattern provides only partial support for H3 and reveals a partially unexpected differentiation between cyber and face-to-face IPV: rather than a clean gradient in which lower empathy corresponds to more violence, the Dark Cognitive Empath profile combines preserved cognitive empathy with a level of cyber monitoring/control comparable to the Dark Non-Empathic profile, while the Dark Non-Empathic profile remains the more consistent driver of face-to-face aggression. However, given the small size of the Dark Cognitive Empath profile, this interpretation should be regarded as preliminary and requires replication.

Previous research on cyber IPV provides a possible explanation for this pattern. Cyber channels can reduce exposure to the target’s immediate emotional and behavioural reactions, while physical distance may weaken the social and affective cues that ordinarily inhibit aggression (Pineda et al., 2022, 2023). Recent research further suggests that the association between dark personality traits and problematic digital behaviour may involve relational processes that are particularly relevant in online contexts. Giancola et al. (2026) found that jealousy and cyberstalking sequentially mediated the association between Dark Tetrad traits and problematic social media use. Although these processes were not assessed in the present study, they provide a useful theoretical framework for understanding how dark traits may be expressed through digitally mediated relational behaviours. Jealousy may contribute to increased surveillance of a partner’s online activity, while the accessibility and persistence of digital information may facilitate cyberstalking and monitoring behaviours. Such processes may be particularly relevant to the monitoring/control dimension of cyber IPV examined here. One possible interpretation is that individuals with preserved cognitive empathy may be able to understand another person’s vulnerabilities and anticipate which actions are likely to cause distress. When this capacity is not accompanied by a correspondingly strong affective response to the other person’s suffering, it may be used instrumentally rather than protectively. However, the present study did not assess whether participants deliberately used cognitive empathy to identify vulnerabilities, manipulate partners, or facilitate abusive behaviour. Given the cross-sectional design of the present study, this instrumental-use interpretation should be understood as one plausible theoretical explanation rather than a mechanism demonstrated by the present findings. To establish whether cognitive empathy is actively deployed in this way would require longitudinal or experimental designs. This interpretation is consistent with research showing that cognitive and affective empathy represent distinguishable processes and that dark traits are more consistently associated with deficits in affective empathy than with an inability to understand others’ perspectives (Heym et al., 2019; Wai & Tiliopoulos, 2012). Cyber contexts may facilitate this pattern by removing physical presence, immediate visible distress, and other situational cues that could otherwise discourage aggressive behaviour.

Our findings are consistent with the possibility, raised by Cascardi et al. (2025), that interventions aimed at increasing empathic capacity could paradoxically enhance the manipulative potential of individuals in this profile. Approaches that treat empathy as a uniformly protective construct may overlook individuals who possess strong perspective-taking abilities but use those abilities in manipulative or harmful ways. This possibility is consistent with the distinction between cognitive empathy, as the capacity to understand another person’s mental state and affective empathy, as the tendency to share or respond emotionally to that state (Cuff et al., 2016; Decety & Jackson, 2004). Interventions should therefore avoid assuming that increases in perspective-taking alone will necessarily reduce aggression. For individuals with elevated dark traits and already-preserved cognitive empathy, it may be more useful to combine perspective-taking work with components addressing affective concern, moral disengagement, responsibility for harm, and the use of coercive or manipulative behaviours. The present findings raise the possibility that perspective-taking alone may not necessarily be associated with reduced aggression, although this possibility requires further investigation. Rather, they raise the possibility that, among individuals with elevated dark traits and preserved cognitive empathy, other factors such as affective concern, moral disengagement, responsibility for harm, and the use of coercive or manipulative behaviours may also be relevant. Future studies should examine the potential contribution of these factors and determine whether they have implications for the design of intervention programmes. Assessment and prevention programmes may also benefit from examining cyber and face-to-face perpetration separately, as the present results suggest that the psychological characteristics associated with aggression may differ across these contexts.

Several limitations should be noted. First, the sample was recruited through social networks using convenience and snowball sampling, was skewed toward women (70.4%), and was predominantly based in Spain (82.1%), which limits the generalisability of the findings to other populations and sampling frames. Initiating recruitment through the researchers’ own social networks may have introduced substantial selection bias, as individuals within these networks are likely to share certain sociodemographic and sociocultural characteristics. The subsequent snowball sampling procedure may have further reinforced this bias by extending recruitment to socially connected individuals. Consequently, the sample may not adequately represent the broader populations in terms of characteristics such as educational attainment, age, socioeconomic status, or other sociodemographic factors. This potential lack of representativeness should be considered when interpreting both the characteristics and relative prevalence of the profiles identified in the present study. Second, the cross-sectional design does not allow causal conclusions regarding the direction of the relationship between trait profiles and IPV perpetration. Third, as with any study relying on self-report, responses may have been affected by social desirability or under-reporting. However, this concern may be partly mitigated in the case of dark personality traits, as previous research using anonymous self-report conditions has found that individuals high in these traits tend to respond honestly rather than in a self-favourable manner (Galán et al., 2023). Fourth, the CTS2 Sexual Violence subscale showed extremely low internal consistency in the present sample (α = 0.12). Consequently, this subscale cannot be considered a sufficiently reliable scale measure in the present sample, and results involving this outcome are reported only for transparency and are not interpreted substantively. Relatedly, the narcissism subscale of the Spanish SD3 showed relatively low internal consistency in the present sample (α = 0.65, ω = 0.65), while the reliability of the psychopathy subscale was also modest. This is particularly relevant in the context of the person-centred analyses, because measurement error in the relatively brief trait indicators may influence both the number and shape of the latent profiles extracted. Accordingly, the resulting profiles should be interpreted as configurations based on imperfectly measured observed trait scores rather than as error-free representations of underlying personality constructs. Replication using more comprehensive and reliable measures of the Dark Tetrad traits is therefore warranted. Fifth, Profile 3, provisionally labelled the Dark Cognitive Empath group, comprised only 14 participants. Although this profile showed a high average posterior classification probability and emerged as an interpretable subdivision of the broader dark-trait profile identified in the three-profile solution, its small size remains an important limitation. In particular, the limited number of participants reduces the statistical precision of distal-outcome comparisons involving this group, especially for detecting small or moderate between-profile differences. High classification accuracy does not compensate for this limited sample size. Accordingly, both significant and non-significant pairwise comparisons involving the Dark Cognitive Empath profile should be interpreted cautiously, and non-significant differences should not be taken as evidence of equivalence between profiles. The small profile size may also affect the stability and replicability of the profile itself and its associations with IPV. Therefore, the Dark Cognitive Empath label should be regarded as descriptive and provisional, and replication in larger and more diverse samples is needed before this configuration and its distinctive behavioural correlates can be considered robust.

## 5. Conclusions

Our findings suggest that combining Dark Tetrad traits with cognitive and affective empathy within a person-centred framework reveals meaningful heterogeneity that may be obscured by purely variable-centred approaches. Four distinct profiles emerged—Empathic Non-Dark, Typical, Dark Cognitive Empath, and Dark Non-Empathic—and these profiles differed in both the extent and form of IPV perpetration. The Dark Cognitive Empath profile showed the highest estimated level of cyber monitoring/control behaviour, differing significantly from the two non-dark profiles but not from the Dark Non-Empathic profile, without corresponding differences on the individual face-to-face IPV measures. These findings provide preliminary evidence linking the dark empath configuration to IPV perpetration and suggest that preserved cognitive empathy in the context of elevated dark traits may be associated with elevated cyber monitoring/control behaviour, though this pattern was not exclusive to this profile. However, this interpretation should be treated cautiously given the small size of the Dark Cognitive Empath group. More broadly, the results suggest that empathy should not be regarded as uniformly protective and that assessment and prevention approaches may benefit from distinguishing cognitive from affective empathy, particularly when addressing online forms of IPV. Interventions that focus on perspective-taking should therefore also consider affective concern, moral disengagement, and the potential—though not yet established—instrumental use of social understanding.

## Figures and Tables

**Table 1:** Bivariate correlations and means and standard deviations of the Dark Tetrad traits, empathy dimensions, and IPV perpetration variables.

Variable	*M* (*SD*)	1	2	3	4	5	6	7	8	9	10	11	12
1. Narcissism	16.21 (5.26)												
2. Machiavellianism	17.07 (6.10)	0.40 **											
3. Psychopathy	8.87 (4.93)	0.38 **	0.65 **										
4. Sadism	6.12 (5.19)	0.28 **	0.63 **	0.71 **									
5. Affective Empathy	39.07 (6.51)	−0.25 **	−0.14 *	−0.20 **	−0.27 **								
6. Cognitive Empathy	36.99 (3.73)	0.02	0.03	−0.03	−0.02	0.34 **							
7. CDAQ Direct Aggression	11.48 (1.28)	0.03	0.19 **	0.27 **	0.32 **	−0.02	0.03						
8. CDAQ Monitoring/Control	13.64 (6.35)	0.12 *	0.26 **	0.27 **	0.29 **	0.04	0.13 *	0.39 **					
9. CTS2 Physical Violence	0.86 (2.39)	0.12 *	0.14 *	0.21 **	0.24 **	−0.14 **	0.00	0.22 **	0.30 **				
10. CTS2 Sexual Violence	0.23 (1.00)	0.17 **	0.13 *	0.08	0.21 **	−0.18 **	0.00	−0.07	0.09	0.16 **			
11. CTS2 Psychological Violence	5.32 (5.96)	0.16 **	0.19 **	0.23 **	0.20 **	−0.03	0.11 *	0.21 **	0.39 **	0.40 **	0.47 **		
12. CTS2 Negotiation	23.85 (8.72)	0.09	0.07	0.02	0.04	0.10	0.19 **	0.08	0.25 **	0.24 **	0.15 **	−0.04	
13. Sex		0.09	0.09	0.05	0.11	−0.30 **	−0.32 **	−0.04	−0.14 *	−0.02	0.08	0.02	−0.01

*Note.* * *p* < 0.05. ** *p* < 0.01. Pearson correlations are reported for continuous variables. Point-biserial correlations were calculated for associations involving sex, coded 0 = women and 1 = men. *M* and *SD* are reported for continuous variables only. CDAQ = Cyber Dating Abuse Questionnaire; CTS2 = Revised Conflict Tactics Scales.

**Table 2:** Model fit indices for 1- through 6-profile solutions.

Profiles	AIC	BIC	SSA-BIC	Entropy	VLMR *p*	Adjusted LRT *p*
1	5534.824	5580.193	5542.130			
2	5221.716	5293.550	5233.284	0.786	0.002	0.002
3	5104.136	5202.435	5119.966	0.782	0.257	0.263
**4**	**5012.706**	**5137.470**	**5032.797**	**0.832**	**0.046**	**0.048**
5	4989.546	5140.776	5013.900	0.850	0.269	0.274
6	4977.523	5155.218	5006.139	0.796	0.415	0.425

*Note*. AIC, Akaike Information Criterion; BIC, Bayesian Information Criteria; SSA-BIC, BIC adjusted for sample size; VLRM, Vuong-Lo-Mendel-Rubin; LRT, Likelihood Ratio Test. Bolded values indicate the chosen solution.

**Table 3:** Differences in the Scores Among the Four Profiles in the IPV Perpetration Variables of Interest.

Variable	P1 *M* (*SE*)	P2 *M* (*SE*)	P3 *M* (*SE*)	P4 *M* (*SE*)	Wald χ^2^	Overall *p*
CDAQ Direct Aggression ^†^	11.11 (0.07) ^ab^	11.29 (0.10) ^cd^	13.14 (0.74) ^ac^	11.81 (0.15) ^bd^	26.54	<0.001
CDAQ Monitoring/ Control	11.09 (0.64) ^abc^	13.11 (0.55) ^ade^	19.64 (2.22) ^bd^	15.37 (0.75) ^ce^	28.80	<0.001
CTS2 Physical Violence	−0.03 (0.03) ^ab^	0.56 (0.17) ^ac^	0.95 (0.52)	1.99 (0.41) ^bc^	42.30	<0.001
CTS2 Sexual Violence ^†^	0.00 (0.02) ^ab^	0.19 (0.08) ^a^	0.85 (0.57)	0.34 (0.14) ^b^	13.61	0.003
CTS2 Psychological Violence	3.18 (0.69) ^ab^	4.78 (0.49) ^c^	7.20 (1.14) ^a^	7.43 (0.82) ^bc^	20.43	<0.001
CTS2 Negotiation	23.34 (1.30)	24.15 (0.77)	23.26 (1.96)	23.74(1.05)	0.37	0.947

*Note*. P1 = Empathic Non-Dark (*n* = 56); P2 = Typical (*n* = 166); P3 = Dark Cognitive Empath (*n* = 14); P4 = Dark Non-Empathic (*n* = 88). Values are BCH-adjusted *M* (*SE*). Within each row, means sharing the same superscript letter differ significantly in pairwise comparisons (*p* < 0.05); means that do not share a superscript letter do not differ significantly. ^†^ CDAQ Direct Aggression and CTS2 Sexual Violence are retained for consistency and transparency but are not substantively interpreted because of their low internal consistency. BCH-adjusted means may occasionally fall slightly outside the observed score range because of the weighting used to correct for classification uncertainty.

## Data Availability

The anonymized data and analysis materials supporting the findings of this study are publicly available on the Open Science Framework (OSF) at https://osf.io/a57wg/overview (accessed on 24 September 2026).

## Abbreviations

The following abbreviations are used in this manuscript:

IPV	Intimate Partner Violence
TLA	Three-letter acronym
